# Combined identification of *ARID1A*, *CSMD1*, and *SENP3* as effective prognostic biomarkers for hepatocellular carcinoma

**DOI:** 10.18632/aging.202586

**Published:** 2021-02-07

**Authors:** Yuanyuan Zhao, Bo Yang, Dong Chen, Xiaojun Zhou, Meixi Wang, Jipin Jiang, Lai Wei, Zhishui Chen

**Affiliations:** 1 Institute of Organ Transplantation, Tongji Hospital, Tongji Medical College, Huazhong University of Science and Technology, Wuhan 430030, China; 2 Key Laboratory of Organ Transplantation, Ministry of Education, Wuhan 430030, China; 3 NHC Key Laboratory of Organ Transplantation, Wuhan 430030, China; 4 Key Laboratory of Organ Transplantation, Chinese Academy of Medical Sciences, Wuhan 430030, China

**Keywords:** differentially expressed genes, characteristic molecular heterogeneity, population specific biomarkers, hepatocellular carcinoma, HBV infection

## Abstract

Background: The current study aimed to understand the genetic landscape and investigate the diagnostic and prognostic biomarkers of primary hepatocellular carcinoma (HCC).

Methods: A cohort of 36 Chinese HCC samples with hepatitis B virus (HBV) infection was examined by whole-exome sequencing (WES). Prognosis-related alterations were identified and further verified in the TCGA database and GSE65372 profiles in the GEO database. A Chinese replication cohort of 180 HCC samples with HBV infection was collected to evaluate the candidate genes by immunohistochemical analysis. A receiver operating characteristic (ROC) curve analysis evaluated the prognostic power of candidate genes. Finally, EdU and transwell invasion assay were performed to detect the function of candidate genes.

Results: A total of 11 novel genes showed a significant association with HCC in the discovery cohort. The data were verified using the GEO and TCGA databases, and the expression of ARID1A, CSMD1, and SENP was evaluated in the replication cohort. Furthermore, ARID1A, CSMD1, and SENP3 are effective prognostic biomarkers for HCC patients in the replication population.

Conclusions: Molecular heterogeneity was detected in HCC patients, and ARID1A, CSMD1, and SENP3 were identified as effective HCC prognosis biomarkers. CSMD1 prevents HCC by suppressing cell invasion.

## INTRODUCTION

Primary liver carcinoma (PLC) is one of the most frequently occurring cancers worldwide, with >500,000 new cases recorded every year [1], and is the third most common cause of cancer-related deaths [2]. The most frequent type of PLC in adults is hepatocellular carcinoma (HCC), which accounts for >80% of the occurrences. Chronic hepatitis B virus (HBV) infection is the most crucial cause of HCC in China. Furthermore, individuals with HCC do not show any symptoms during the early stage, resulting in late-stage diagnosis, unsatisfactory treatments, and poor prognosis. Over the last decade, our understanding of the genetic predisposition for PLC, especially HCC, has improved significantly [3]. High-throughput analysis of large HCC samples has provided a landscape of HCC genetic alterations at multiple levels, including DNA, transcriptional mRNA, and non-coding RNA [4, 5]. Based on this information, clinical DNA sequencing is considered an essential part of HCC treatment. Therefore, knowledge of the HCC gene landscape is not only imperative to our understanding of HCC heterogeneity but also to explore effective diagnostic and prognostic HCC biomarkers [3, 6, 7].

Although previous studies have identified specific HCC risk factors, such as *CTNNB1, TP53, AXIN1,* and *CNKN2A* [8, 9], the most prevalent diagnostic and prognostic genes for HCC are yet unknown [3]. The present study investigated the diagnostic and prognostic biomarkers of HCC patients in the Chinese population by comprehensive analysis of gene variations and expression in a discovery cohort of 36 HCC samples with HBV-infection and a replication cohort of 180 HCC samples. The gene landscapes of HCC patients with HBV infection were identified, and the resulting prognostic genes were evaluated.

## RESULTS

### Genetic mutation landscape of HCC in the discovery population

**Figure 1:**
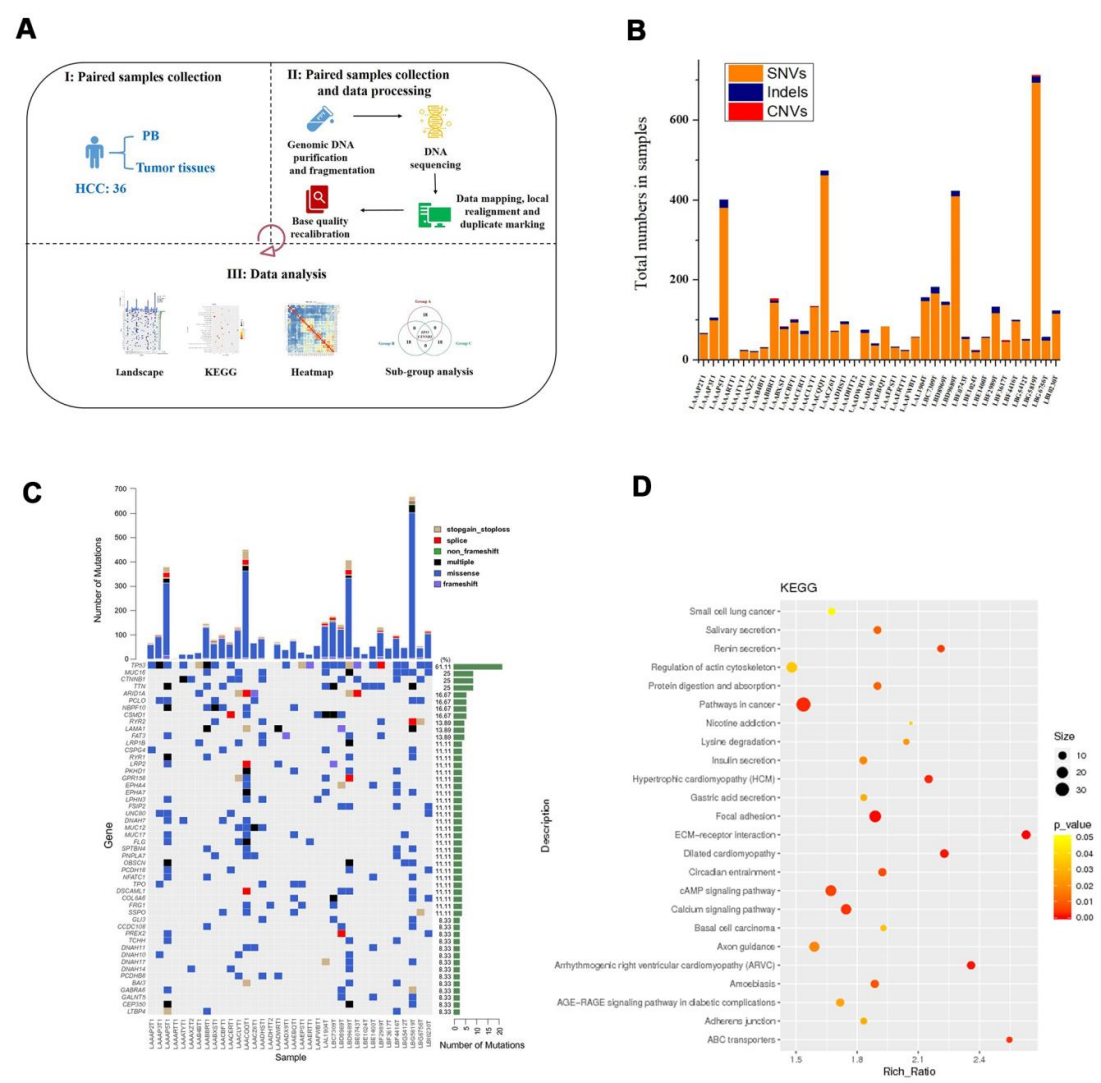
**Process for determining the genetic mutation landscape of HBV-infected HCC cases in the discovery population.** (**A**) Illustration of sequencing data analysis. (**B**) Mean number of SNVs, indels, and CNVs in HCC. (**C**) The most frequently mutated genes in HCC patients. (**D**) KEGG enrichment analysis of genes with mutations in HCC patients.

Patient clinical characteristics are presented in Table 1. Briefly, all subjects were males with an average age of 48.33 ± 9.75 years. All patients had a history of HBV infection. The sample collection, sequencing, and data analysis are shown in Figure 1A. To identify the landscape of genetic mutations of HCC patients with HBV infection, WES was performed in 36 pairs of HCC samples; a total of 4231 somatic SNVs, 192 somatic indels, and 12 somatic CNVs were detected. The mean number of SNVs, indels, and CNVs in each patient was 117.5, 5.3, and 0.3, respectively (Figure 1B). According to the frequency of sample mutations, 8 genes (*TP53, MUC16, CTNNB1, TTN, ARID1A, PCLO, NBPF10,* and *CSMD1*) were identified with a high mutation frequency (> 15%) in HCCs (Figure 1C). *TP53* is the most frequently occurring gene (61.1%). Compared to the most frequently mutated genes in HCC from the TCGA database, *NBPF10* and *CSMD1* were highly mutated genes in HCCs in the Chinese population. Furthermore, KEGG pathway analysis (*P* < 0.05, Figure 1D) identified representative HCC proteins and showed that the calcium signaling pathway, axon guidance, circadian entrainment, and nicotine addiction pathways were significantly enriched in HCC samples.

**Table 1:** Clinical characteristics of HCC patients in the discovery population.

**Sample ID**	**Age at diagnosis (years)**	**Sex**	**Type of LPC**^#^	Group^*^	**Total SNVs**	**Total InDels**	**Total CNVs**
LAAAP2T1	50	male	HCC, HBV-infected	B	65	2	0
LAAAP3T1	49	male	HCC, HBV-infected	B	100	6	0
LAAAP5T1	56	male	HCC, HBV-infected	B	381	20	0
LAAARTT1	51	male	HCC, HBV-infected	B	3	0	0
LAAATYT1	54	male	HCC, HBV-infected	C	23	1	0
LAAAXZT2	53	male	HCC, HBV-infected	B	21	1	0
LAAB4BT1	57	male	HCC, HBV-infected	A	30	1	0
LAABBRT1	43	male	HCC, HBV-infected	NA	144	6	4
LAABXST1	39	male	HCC, HBV-infected	C	78	5	1
LAACBFT1	59	male	HCC, HBV-infected	C	95	6	1
LAACERT1	47	male	HCC, HBV-infected	A	65	8	0
LAACLYT1	56	male	HCC, HBV-infected	C	133	1	1
LAACQQT1	60	male	HCC, HBV-infected	NA	462	12	0
LAACZ6T1	27	male	HCC, HBV-infected	A	71	1	0
LAADHST1	45	male	HCC, HBV-infected	B	91	5	0
LAADHTT2	60	male	HCC, HBV-infected	B	1	0	0
LAADWRT1	36	male	HCC, HBV-infected	A	69	6	0
LAADX9T1	34	male	HCC, HBV-infected	C	37	4	0
LAAEBQT1	49	male	HCC, HBV-infected	B	84	0	0
LAAEPST1	25	male	HCC, HBV-infected	B	31	2	0
LAAERTT1	36	male	HCC, HBV-infected	B	23	2	0
LAAFWBT1	49	male	HCC, HBV-infected	B	57	1	0
LAL1904T	67	male	HCC, HBV-infected	NA	148	9	0
LBC7309T	62	male	HCC, HBV-infected	NA	168	15	0
LBD8969T	49	male	HCC, HBV-infected	NA	139	6	0
LBD9689T	56	male	HCC, HBV-infected	NA	410	13	0
LBE0743T	42	male	HCC, HBV-infected	A	53	4	0
LBE1024T	34	male	HCC, HBV-infected	NA	21	3	1
LBE1400T	49	male	HCC, HBV-infected	A	56	1	0
LBF2989T	53	male	HCC, HBV-infected	NA	118	15	0
LBF3617T	54	male	HCC, HBV-infected	NA	46	2	1
LBF4416T	50	male	HCC, HBV-infected	NA	98	2	0
LBG5412T	55	male	HCC, HBV-infected	NA	49	3	0
LBG5819T	51	male	HCC, HBV-infected	A	695	15	3
LBG6756T	43	male	HCC, HBV-infected	A	49	8	0
LBI0230T	40	male	HCC, HBV-infected	A	117	6	0

HCC, hepatocellular carcinoma.

^*^Group A: HCCs with tumor recurrence within 6 months after LT. Group B: HCCs without tumor recurrence > 1 year after LT. Group C: HCCs with tumor recurrence > 1 year after LT.

### Prognosis-related alterations in the discovery population

**Figure 2:**
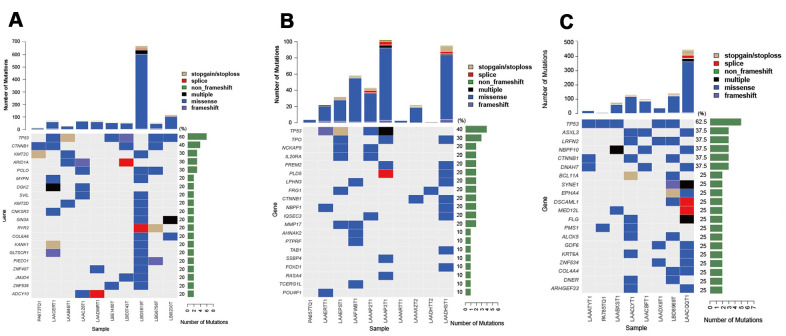
**Top 10 most frequently mutated genes in groups A–C of HCC patients with different prognostic impact.** (**A**) Top 10 frequently mutated genes in group A (n = 10, recurrence within 6 months after LT). (**B**) Top 10 frequently mutated genes in group B (n = 10, non-recurrence for > 1 year after LT). (**C**) Top 10 frequently mutated genes in group C (n = 8, recurrence more than a year after LT).

To evaluate the prognostic impact of mutated genes HCC patients were divided into three groups: recurrence within 6 months after undergoing LT (group A, n = 10), non-recurrence for > 1 year after undergoing LT (group B, n = 10), and recurrence > 1 year after undergoing LT (group C, n = 8). The top 10 most frequently mutated genes of each group (A, B, and C) are presented in Figure 2A–2C. Compared to patients without tumor recurrence > 1 year after LT (Group B), patients with tumor recurrence within 6 months had significantly different gene landscapes. Only *TP53* and *CTNNB1* genes were highly and frequently mutated between the two groups. In addition, the types of variations were complicated in patients with early recurrence. These results demonstrated that significant genetic heterogeneity affected HCC prognosis.

To further identify the representative genes associated with HCC prognosis, alterations in the five most frequently mutated genes (*TP53, CTNNB1, KMT2C, ARID1A,* and *PCLO*) were detected between groups A and B. *CTNNB1, KMT2C, ARID1A*, and *PCLO* were significantly associated with HCC prognosis (likelihood ratio *P* = 0.018 for *CTNNB1* and likelihood ratio *P* = 0.03 for *KMT2C, ARID1A*, and *PCLO*; Table 2). Furthermore, we also compared the expression of these five genes between tumor samples (n = 39) and non-tumor samples (n = 15) based on the expression dataset GSE65372. These findings revealed that *KMT2C* and *ARID1A* were significantly and differentially expressed between the two groups (adjusted *P*-values = 0.046 and 0.039, respectively, Table 2). Therefore, it could be deduced that *KMT2C* and *ARID1A* are significantly associated with HCC prognosis.

**Table 2:** Comparison of genes associated with HCC prognosis in groups A and B^#^.

**Gene**	**Frequency in group A**	**Frequency in group B**	**Likelihood ratio *P*-value in the discovery population**	**Adjusted *P*-value in GEO database**
*TP53*	0.6	0.40	>0.05	>0.05
*CTNNB1*	0.4	0.20	0.018^*^	>0.05
*KMT2C*	0.3	0	0.03^*^	0.046^*^
*ARID1A*	0.3	0	0.03^*^	0.039^*^
*PCLO*	0.3	0	0.03^*^	>0.05

^#^Group A: HCCs with tumor recurrence within 6 months after LT; Group B: HCCs without tumor recurrence > 1 year after LT; ^*^P-value < 0.05.

### New HCC driver genes identified in the discovery population

Herein, we attempted to discover new driver genes of HCC in patients with early and late tumor recurrence, using the CHASM software to identify the driver genes in HCC. Finally, except for the known driver genes of HCC, five new driver genes of HCC, including *MAP4K3, COX5B, ACTN3, CFTR*, and *LRRC7,* were associated with the early recurrence of HCC (recurrence within 6 months) and 2 genes, *PRKCG* and *SENP3,* were considered new driver genes associated with the late recurrence of HCC (recurrence after 1 year) (*P* < 0.005, false discovery rate (FDR) ≤ 0.1, Table 3). These results showed that the driver genes differed significantly between patients with early and late tumor recurrence.

**Table 3:** New driver genes associated with early-recurrence and late-recurrence of HCC.

**Group**^#^	**Gene**	**CHASM Score**	***P*-value**	**FDR**
A	*MAP4K4*	0.738	1.20E-03^*^	0.05
A	*COX5B*	0.728	1.80E-03^*^	0.1
A	*ACTN3*	0.712	2.20E-03^*^	0.1
A	*CFTR*	0.698	3.80E-03^*^	0.1
A	*LRRC7*	0.696	4.00E-03^*^	0.1
C	*PRKCG*	0.68	1.72E-04^*^	0.05
C	*SENP3*	0.744	8.00E-04^*^	0.1

^#^Group A: HCCs with tumor recurrence within 6 months after LT; Group C: HCCs with tumor recurrence > 1 year after LT; ^*^P-value < 0.05.

### ARID1A, CSMD1, and SENP3 are associated with poor prognosis in HCC from TCGA database

As described above WES and comparative analysis identified 11 novel genes (*KMT2C*, *ARID1A, NBPF10*, *CSMD1, MAP4K3, COX5B, ACTN3, CFTR*, *LRRC7, PRKCG*, and *SENP3*) that were significantly associated with HCC in our Chinese population. To validate these findings, the expression (Supplementary Figure 1) and survival (Supplementary Figure 2) patterns of these genes were observed in the TCGA database. Compared to the control samples, three genes (*ARID1A, CAMD1*, and *SENP3*) showed significantly high expression in HCC patients (*P* < 0.05, Supplementary Figure 1B, 1E, 1J) and were associated with poor prognosis (*P* < 0.05, Supplementary Figure 2B, 2E, 2J). According to the TCGA database, the data of *KMT2C* expression were insufficient, while the remaining seven genes did not show any association with HCC prognosis (*P* > 0.05, Supplementary Figure 2A, 2C, 2D, 2F–2I).

### ARID1A, CSMD1, and SENP3 are effective prognostic biomarkers for HBV-infected HCC patients in the replication population

**Figure 3:**
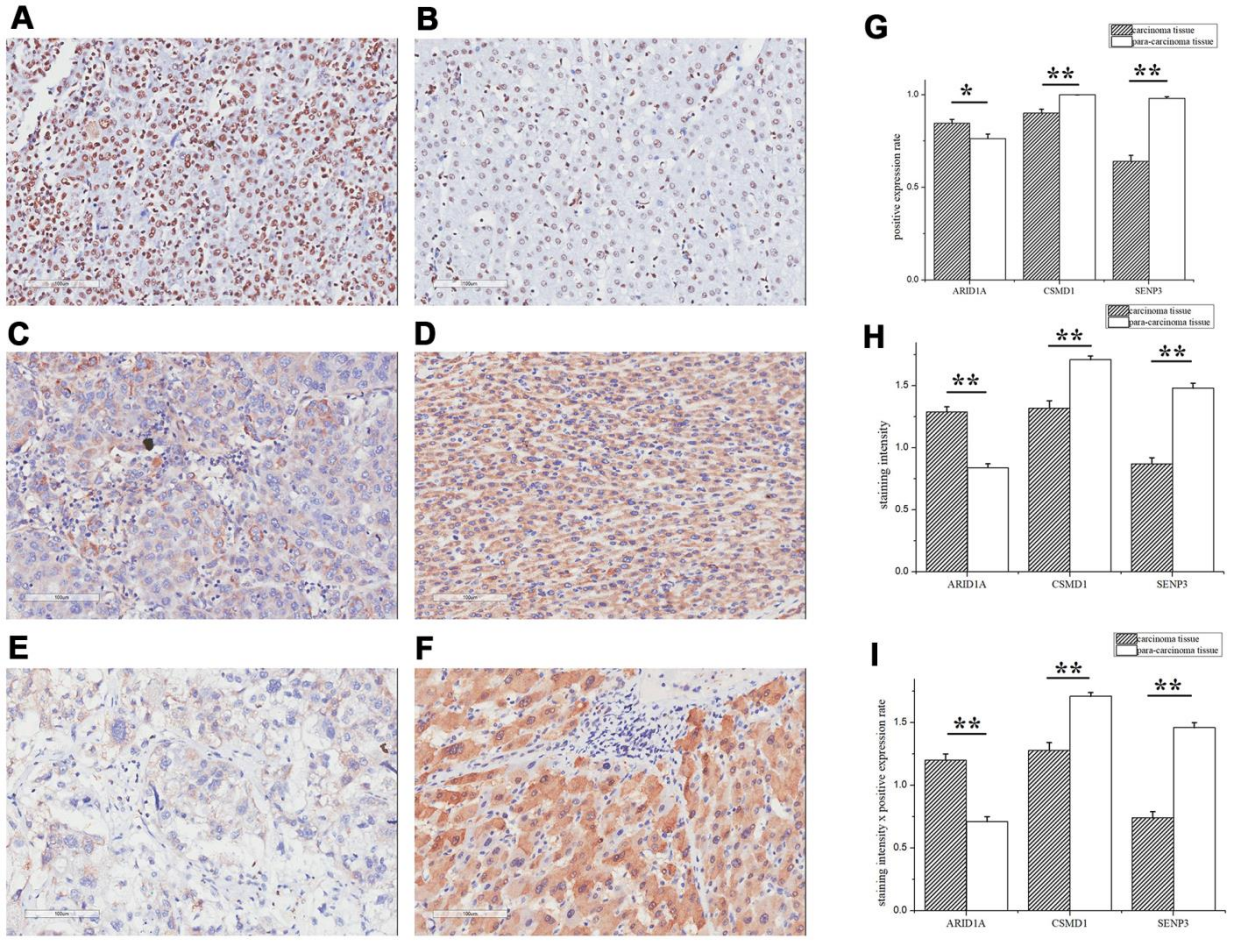
**ARID1A, CSMD1, and SENP3 expression differed significantly between tumor and paracarcinoma tissues.** The IHC assay images of ARID1A (**A**, **B**), CSMD1 (**C**, **D**), and SENP3 (**E**, **F**) expression. Left images are tumor tissues (**A**, **C**, **E**) and right images are paracarcinoma tissues (**B**, **D**, **F**). (**G**) The positive expression rates of ARID1A, CSMD1, and SENP3 in tumor and paracarcinoma tissues. (**H**) The staining intensity of ARID1A, CSMD1, and SENP3 in tumor and paracarcinoma tissues. (**I**) The staining intensity × positive expression rate of ARID1A, CSMD1, and SENP3 in tumor and paracarcinoma tissues. Immunofluorescence staining, ×200. A paired-samples t-test was performed to test the difference between tumor and paracarcinoma tissues. Data are shown as mean ± SD. **, *P* < 0.05.

Herein, we focused on *ARID1A, CSMD1,* and *SENP3* during tissue microarray analysis of 180 HCC samples paired with tumor and paracarcinoma tissues as a replication population. These three genes showed significantly high expression in HCC patients in the TCGA database. The clinical characteristics of these patients are presented in Table 4. The IHC analysis of ARID1A, CSMD1, and SENP3 proteins showed that the expression level of all the three genes was significantly different between tumor and paracarcinoma tissues (*P* < 0.05, Figure 3G–3I). The ARID1A expression was significantly increased in tumor tissues (*P* < 0.05, Figure 3A, 3B) consistent with the TCGA database. Surprisingly, the expression of CSMD1 and SENP3 was significantly decreased in tumor tissues (*P*-value < 0.05, Figure 3C, 3D for CSMD1, and Figure 3E, 3F for SENP3).

**Table 4:** Clinical characteristics of the discovery population.

**Parameters**	**Validation samples for HCC (n = 175)**
Sex (Number of males, %)	149, 85.14%
Age (years mean ± SD)	52.65 ± 11.26
Number of tumors (mean ± SD)	1.18 ± 0.48
Size of the tumor (cm, mean ± SD)	5.20 ± 3.25
Total bilirubin (μmol/L, mean ± SD)	14.37 ± 6.10
ALT (U/L, mean ± SD)	57.70 ± 84.01
ALB (g/dL, mean ± SD)	4.36 ± 0.52
AFP (μg/L, mean ± SD)	4846.82 ± 14002.93
GGT (U/L, mean ± SD)	82.37 ± 77.46
Liver cirrhosis (number, %)	154, 88.00%
AJCC staging (Version VII)	
Stage 1 (number, %)	116, 66.29%
Stage 2 (number, %)	57, 32.57%
Stage 3 (number, %)	2, 1.14%
Pathology grade	
Grade I (number, %)	1, 0.57%
Grade I-II (number, %)	5, 2.86%
Grade II (number, %)	94, 53.71%
Grade II-III (number, %)	28, 16.00%
Grade III (number, %)	47, 26.86%

ALT: Alanine transaminase; ALB: Albumin; AFP: Alpha-fetoprotein; GGT: Glutamine transferase.

**Figure 4:**
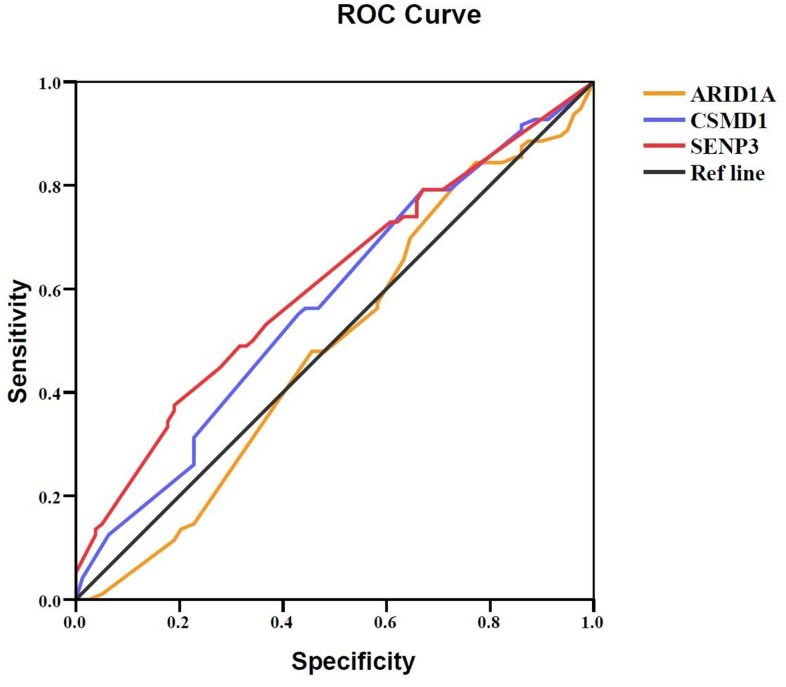
**Prognostic value of ARID1A, CSMD1, and SENP3 expression in HCC tissues.** A ROC curve analysis was performed to evaluate the prognostic power of the genes. *ARID1A, CSMD1*, and *SENP3* yielded AUC values of 0.489 (95% CI: 0.402–0.576; *P* > 0.05), 0.573 (95% CI: 0.488–0.658; *P* > 0.05), and 0.609 (95% CI: 0.526–0.692; *P* = 0.013), respectively. *P* < 0.05 indicates statistical significance.

Furthermore, ROC curve analysis assessed the prognostic power of the three genes based on their expression levels: intensity × positive expression rate. Only *SENP3* had significant diagnostic power (AUC = 0.609, *P* = 0.013, Table 5 and Figure 4), while *ARID1A* and *CSMD1* showed weak diagnostic power (AUC = 0.489 for *ARID1A* and 0.573 for *CSMD1*; *P* > 0.05; Table 5 and Figure 4). Cox regression analysis evaluated the prognostic power of the three genes (Table 6) and showed that *SENP3* as a single gene, the combination of two genes (*ARID1A* and *SENP3*; *CSMD1* and *SENP3*), and the combination of three genes (*ARID1A, CSMD1,* and *SENP3*) could be used as prognostic biomarkers for HCC (*P* < 0.05, Table 6 and Figure 5). Among these, the combination of *CSMD1* and *SENP3* genes was the optimal prognostic biomarker for HCC in our replication population (*P* = 0.006, Table 6 and Figure 5). Moreover, the expression level of *SENP3* ≤ 1.2 and *CSMD1* < 1.5 was significantly associated with poor HCC prognosis. Based on these results, *ARID1A*, *CSMD1,* and *SENP3*, especially the combination of *CSMD1* and *SENP3*, are effective prognostic biomarkers for HCC individuals in the Chinese population.

**Table 5:** ROC curve analysis of ARID1A, CSMD1, and SENP3 expression in HCC survival prediction.

**Parameters**	**Value range**	**Cutoff value**	**AUC**	**SE**	***P*-value**	**95% CI**
ARID1A	0–2.5	1.5	0.489	0.045	0.803	0.402–0.576
CSMD1	0–3	1.5	0.573	0.043	0.099	0.488–0.658
SENP3	0–2.5	1.2	0.609	0.042	0.013^*^	0.526–0.692

AUC: Area under the curve; SE: Standard error; CI: Confidence interval.

^*^P-value < 0.05.

**Table 6:** Prognostic value of ARID1A, CSMD1, and SENP3 expression in HCC using the Cox regression model.

**Parameters**	**Group 1 value**	**Group 2 value**	***P***_adj_ **value**	**HR (95% CI)**
*ARID1A*	≤1.5	>1.5	0.191	0.702 (0.413–1.194)
*CSMD1*	<1.5	≥1.5	0.125	1.425 (0.906–2.241)
*SENP3*	≤1.2	>1.2	0.032^*^	1.858 (1.054–3.272)
*ARID1A* and *CSMD1*	≤1.5 for *ARID1A* and ≥1.5 for *CSMD1*	>1.5 for *ARID1A* or <1.5 for *CSMD1*	0.218	0.685 (0.375–1.251)
*ARID1A* and *SENP3*	≤1.5 for *ARID1A* and >1.2 for *SENP3*	>1.5 for *ARID1A* or ≤1.2 for *SENP3*	0.038^*^	0.554 (0.317–0.969)
*CSMD1* and *SENP3*	≥1.5 for *CSMD1* and >1.2 for *SENP3*	<1.5 for *CSMD1* or ≤1.2 for *SENP3*	0.006^*^	0.535 (0.342–0.837)
*ARID1A* and *CSMD1* and *SENP3*	≤1.5 for *ARID1A* and ≥1.5 for *CSMD1* or >1.2 for *SENP3*	>1.5 for *ARID1A* or <1.5 for *CSMD1* or ≤1.2 for *SENP3*	0.046^*^	0.528 (0.282–0.989)

^*^P-value < 0.05.

**Figure 5:**
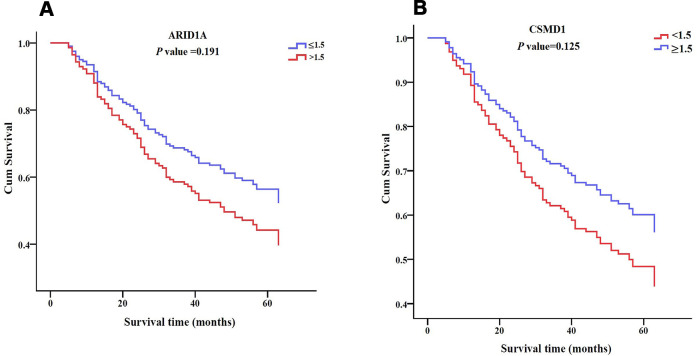
**Survival patterns of ARID1A, CSMD1, and SENP3 expression in HCC tissues.** (**A**) Survival pattern of *ARID1A* expression. *P* > 0.05. (**B**) Survival pattern of *CSMD1* gene. *P* > 0.05.

**Figure 5:**
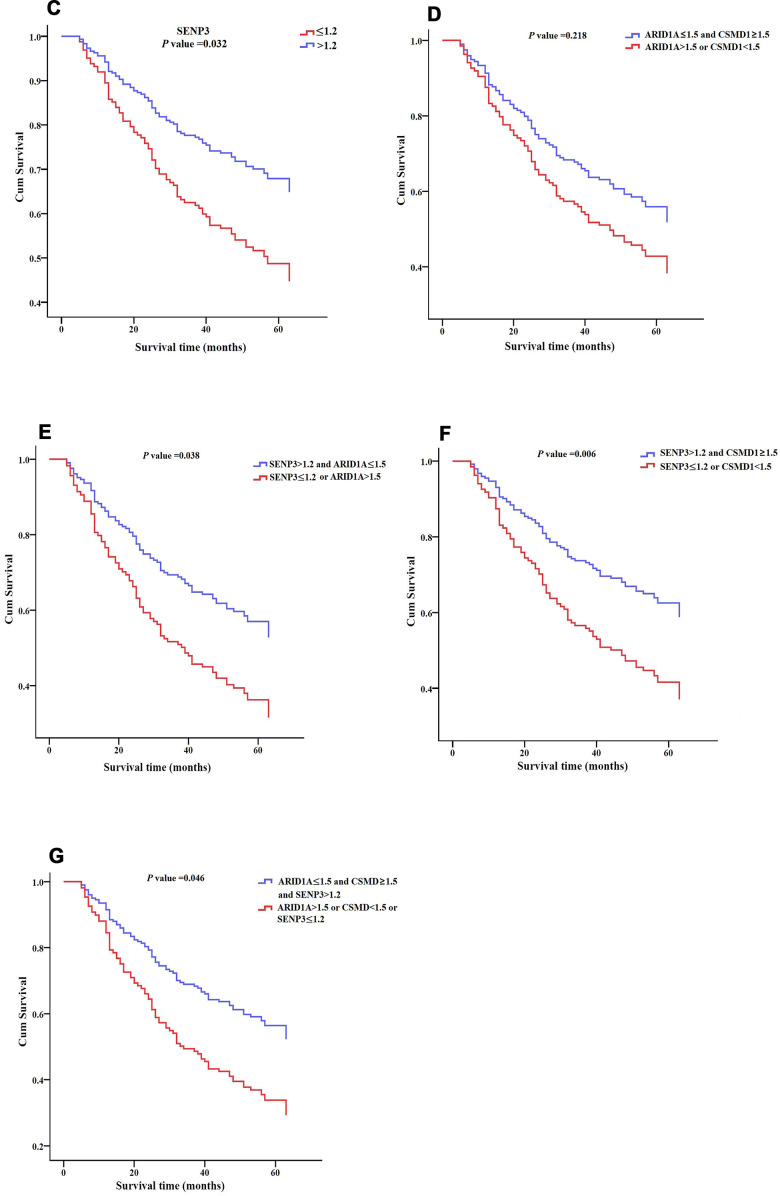
**Survival patterns of ARID1A, CSMD1, and SENP3 expression in HCC tissues.** (**C**) Survival pattern of *SENP3* expression. *P* = 0.032. (**D**) Survival pattern of combined *ARID1A* and *CSMD1* expression. *P* > 0.05. (**E**) Survival pattern of combined *ARID1A* and *SENP3* expression. *P* = 0.038. (**F**) Survival pattern of combined *CSMD1* and *SENP3* expression. *P* = 0.006. (**G**) Survival pattern of combined *ARID1A*, *CSMD1*, and *SENP3* expression. *P* = 0.046. *P* < 0.05 indicates statistical significance.

### CSMD1 prevented HCC by suppressing cell invasion

**Figure 6:**
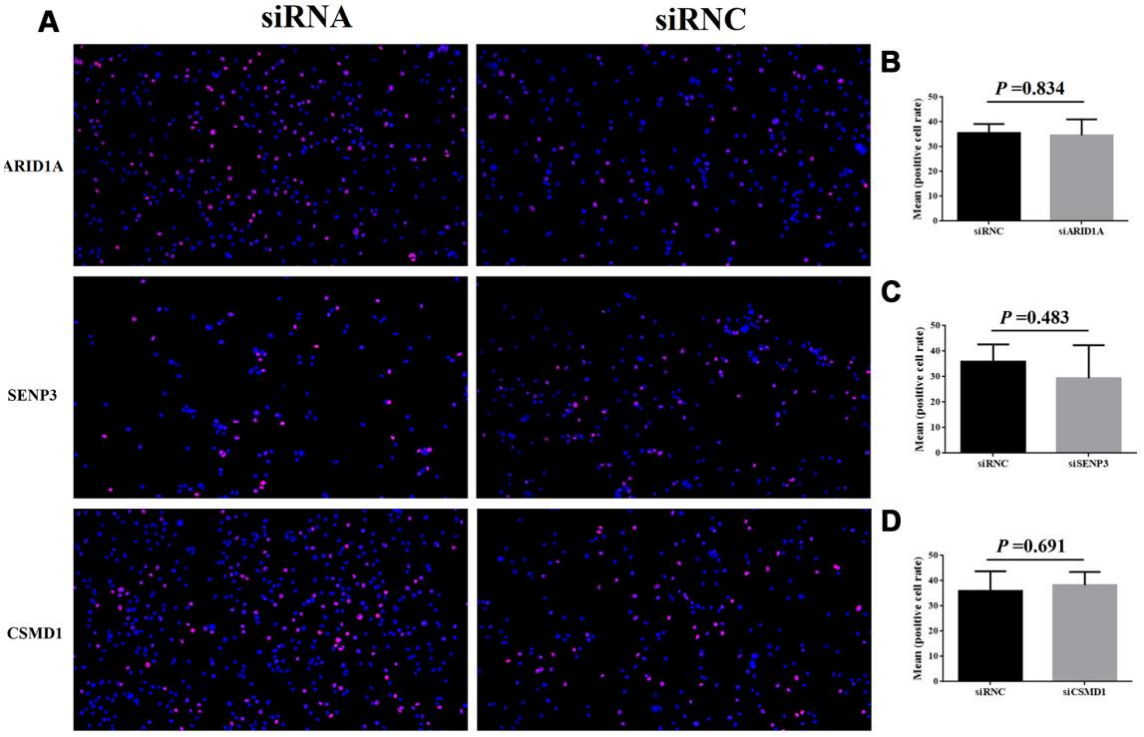
**Cell proliferation after the suppression of genes, ARID1A, CSMD1, and SENP3 by siRNA.** The images of EdU analysis shown in (**A**) were scanned, quantified, and plotted in (**B**–**D**, respectively).

**Figure 7:**
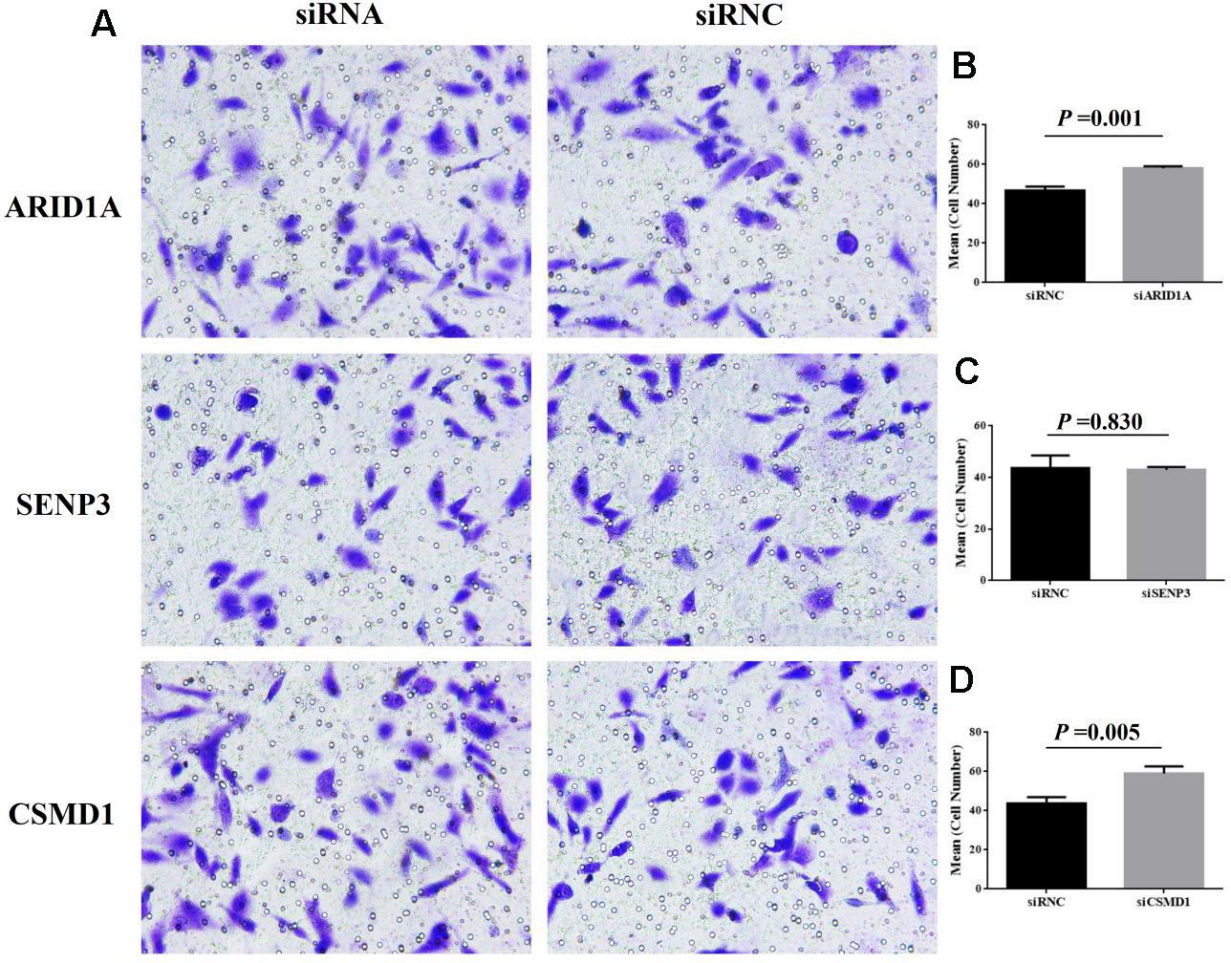
**Cell invasion after the suppression of genes, ARID1A, CSMD1, and SENP3 by siRNA.** The images of transwell invasion assay shown in (**A**) were scanned, quantified, and plotted in (**B**–**D**, respectively).

Finally, to verify the function of *ARID1A, SENP3,* and *CSMD1* genes, we performed cell proliferation and invasion assays in the hepatic Hep3B cell line. In the EdU assay, after the suppression of *ARID1A, SENP3,* and *CSMD1* genes by siRNAs, respectively, for 48 h, the cell proliferation rate was slightly increased by suppression of *CSMD1* (Figure 6A, 6D, *P* > 0.05), slightly reduced by suppression of *SENP3* (Figure 6A, 6C, *P* > 0.05), and almost unaltered by suppression of *ARID1A* (Figure 6A, 6B, *P* > 0.05). On the other hand, transwell invasion assay revealed that the cell invasion rate was unaltered by the suppression of *SENP3* (Figure 7A, 7C, *P* > 0.05) but significantly increased by the suppression of *CSMD1* (Figure 7A, 7D, *P* < 0.05), which was consistent with the results of tissue microarray analysis. Also, the suppression of *ARID1A* elevated the cell invasion rate (Figure 7A, 7B, *P* < 0.05), which was inconsistent with the results of tissue microarray analysis and TCGA database. Therefore, based on these functional assays, we deduced that CSMD1 prevented HCC by suppressing cell invasion.

## DISCUSSION

Recent studies have focused on the genetic characteristics of HCC and several diagnostic and prognostic biomarkers, including genes, non-coding RNAs, and specific factors, such as telomere length [10–12]. Since the molecular characteristics of HCC are not clearly understood, the present study analyzed the genetic landscape of HCC cases with HBV infection in the Chinese population. This study identified several novel candidate and driver genes associated with HCC, and provided evidence that these genes are significantly related to the prognosis of early-recurrence and late-recurrence HCC using the GEO and TCGA databases. We also determined that *ARID1A*, *CSMD1*, and *SENP3* (especially *CSMD1* and *SENP3* combined) genes are effective prognostic biomarkers for HCC in an independent replication population.

Compared to a variety of carcinomas, such as lung carcinomas, a high level of characteristic molecular heterogeneity exists in HCC that could be attributed to complex genetic and epigenetic factors [13, 14]. Based on the current results, the abundance and diversity of variants are observed in HCC patients (the mean number of SNVs, indels, and CNVs was 117.5, 5.3, and 0.3, respectively). Among these gene mutations, very few occurred frequently (*TP53, MUC16, CTNNB1, TTN, ARID1A, PCLO, NBPF10*, and *CSMD1*), which might explain the relatively poor prognosis and insufficient effective target-drug treatment for HCC. *TP53* is the most frequently mutated gene in cancer with tumor suppressor functions [15]. Moreover, *NBPF10* and *CSMD1* are novel and the most frequently mutated genes in HCC individuals with HBV infection, indicating that chronic liver injury, such as HBV infection, affects molecular heterogeneity by interacting with host DNA.

Driver mutations and genes in cancers confer a selective advantage, which differs from the coexistence of passenger mutations in successfully expanded clonal cell lines [16, 17]. Thus, identifying new driver genes is one of the greatest challenges in cancer genetics [18]. Critical driver mutations and genes that contribute to the understanding of molecular pathogenesis of HCC have been identified previously [19]. Notably, many new driver genes were associated with the early (within 6 months) and late recurrence of HCCs (recurrence after 1 year) (*P*-value < 0.005, FDR ≤ 0.1, Table 3) in those with HCC and HBV infection, suggesting that HBV-derived processes are associated with specific mutational signatures.

*ARID1A* has been reported in HCC in Asians [20]. Sun et al. speculated that *ARID1A* exerts a tumor-suppressive (in progression and metastasis) and oncogenic (in primary tumors) role in HCC [21]. Herein, we also observed that high *ARID1A* expression was significantly associated with poor HCC prognosis. Surprisingly, the expression of CSMD1 and SENP3 was significantly reduced in tumor tissues (*P*-value < 0.05, Figure 3C, 3D for CSMD1 and Figure 3E, 3F for SENP3), which is opposite to the information provided by the TCGA database. Typically, *CSMD1* is a tumor suppressor gene that encodes CUB and sushi domain-containing protein-1 (CSMD1). Zhu et al. [22] observed that decreased protein expression of CSMD1 significantly promoted HCC cell proliferation, migration, and invasion, suggesting its functional role as a tumor suppressor gene in HCC. Furthermore, we identified that CSMD1 prevents HCC by suppressing the cell invasion *in vitro*. Moreover, SENP3 plays a critical role in increasing the stability of tumor suppressor P53 protein by attenuating Mdm2-mediated p53 ubiquitination and degradation [23]. Furthermore, *SENP3* gene-encoded stress-sensitive SUMO-2/3-specific peptidase contributes to a host defense mechanism by restoring host protein translation and suppressing *HBV* gene expression in HBV infection [24]. Therefore, it could be hypothesized that the reduced expression of CSMD1 and SENP3 proteins is associated with HCC recurrence, progression, and poor prognosis. These findings need to be substantiated using large samples in future studies.

Nevertheless, the present study has several limitations. First, the number of sequenced samples was small (36 HCC samples), necessitating the sequencing of additional samples to establish a comprehensive genomic landscape for HCC patients with HBV infection in the Chinese population. Furthermore, we found that the effect of *SENP3* and *ARID1A* genes was inconsistent in different assays. Therefore, additional functional studies are required to verify the molecular mechanisms underlying *SENP3* and *ARID1A* genes in HCC.

## MATERIALS AND METHODS

### Clinical samples

For the discovery population, a total of 36 HCC samples were collected from patients who underwent liver transplant (LT) in our center between 2017 and 2018. All liver grafts were voluntarily donated after death, and informed consent was obtained from all recipients before LT. Tumor tissues obtained from each patient were fixed with 4% paraformaldehyde and embedded in paraffin. Additionally, 10 mL peripheral blood (PB) was collected from each case-matched patient for paired-analysis, as described previously [25, 26]. For the replication cohort, a tissue microarray of 180 HCC samples with HBV infection paired with tumor and paracarcinoma tissues (HLivH180Su08 and HLivH180Su15, Shanghai Outdo Biotech Co. Ltd, Shanghai, China) was performed for immunohistochemical (IHC) analysis. HCC samples were diagnosed by histological analysis.

### RNAi, cell culture, and transfection

Hep3B cell line was purchased from ATCC (American Type Culture Collection, Rockville, MD, USA) and cultured in RPMI-1640 medium supplemented with 10% fetal bovine serum (FBS) at 37° C in a humidified incubator with 5% CO_2_. *ARID1A*, *CSMD1*, and *SENP3* shRNAs and siRNC were designed and synthesized by Guangzhou RioboBio (Guangzhou, Guangdong, China). The target sequences are listed in Table 7. For transfection, the cells were seeded at a density of 1×10^5^ cells/well for 24 h. Then, the cells were transfected with siRNA fragments and negative control siRNAs.

**Table 7:** siRNA sequences for ARID1A, SENP3, and CSMD1.

**Name**	**Sequence**
st-h-ARID1A-1	CACCTCTCCTAGCAAGTCT
st-h-ARID1A-2	GATCCTTATGGCAGCATGA
st-h-ARID1A-3	CAGGCAGCCAAACTATAAT
st-h-SENP3-1	GGAGGAGGATGAAGATGAA
st-h-SENP3-2	CCAGCATCCTCATCAGCAA
st-h-SENP3-3	GCAGGACATGCCCAAACTT
st-h-CSMD1-1	GGACAAGCATCGTTTGAAA
st-h-CSMD1-2	GGATGATGATTTCGAAATA
st-h-CSMD1-3	GAACCAAACTACAACATTA

### Whole-exome sequencing (WES) and discovery population analysis

Genomic DNA was extracted from tumor tissues and matched to the PB samples. Genomic DNA libraries were prepared using the protocols provided by Illumina HiSeq2000 platform (Genetron Health Co., Ltd). WES was performed using a TruSeq Exome Enrichment kit (Illumina). The captured DNA libraries were sequenced on an Illumina HiSeq2000 Genome Analyzer. WES provided a 200× average coverage for tumor and PB samples. After raw data (FASTQ) were collected, quality control was performed. The BAM files were processed for local realignment, duplicate removal, and base quality recalibration using GATK (https://software.broadinstitute.org/gatk/) and Picard Tools (http://broadinstitute.github.io/picard/index.html). Mutations were annotated using ANNOVAR [27]. The tumor tissues and normal PB sequencing reads were compared to identify germline single nucleotide variants (SNVs)/insertions-deletions (indels) and somatic SNVs/indels/copy number variants (CNVs)/structural variations (SVs).

### Pathway enrichment analysis

Pathway enrichment analyses for genes harboring SNVs, SVs, or differentially expressed genes (DEGs) were performed using Kyoto Encyclopaedia of Genes and Genomes (KEGG) canonical pathways and DAVID Bioinformatics Resources 6.7 database (Annotation, Visualisation, and Integrated Discovery; https://david-d.ncifcrf.gov/). *P* < 0.05 indicated statistical significance.

### GEO dataset retrieval for HCC analysis

The HCC expression dataset GSE65372 [28] was downloaded from GEO (Gene Expression Omnibus) [29] and analyzed using the GEO2R online analyzer. Next, we identified DEGs and compared the cases (tumor samples, n = 39) and controls (non-tumor samples, n = 15) using the GEO2R online analyzer. Adjusted *P*-values < 0.05 were considered significant, using the Benjamini–Hochberg procedure.

### Identification of new driver genes

In a recent study, we used CHASM software (https://wiki.chasmsoftware.org/) to identify the driver genes in HCC, as described previously [30]. Briefly, CHASM predicts the functional significance of somatic missense mutations, using a Random Forest classifier trained with 49 predictive features. This method classifies the predictive data and provides the corresponding scores. Furthermore, the CHASM method is used to test the hypothesis between the scores and the passenger genes in the random forest training set; finally, the *P*-value is adjusted by Benjamini–Hochberg correction.

### TCGA dataset retrieval for HCC analysis

Gene expression and survival data of somatic mutations were downloaded from the TCGA database [31] and analyzed using the UALCAN analyzer (Analyze, Integrate, Discover; http://ualcan.path.uab.edu/index.html). DEGs and survival patterns with *P*-values < 0.05 between HCC patients and controls in the TCGA database were identified using UALCAN.

### IHC analysis for the replication population

The tissue microarray (HLivH180Su08 and HLivH180Su15, Shanghai Outdo Biotech Co. Ltd, China) was incubated in a dry oven at 63° C for 1 h. Subsequent IHC analysis was performed, as described previously [32]. Briefly, the slides were incubated with primary antibodies (ARID1A (ab182560), 1:10000; CSMD1 (ab198906), 1:4000; and SENP3 (ab247139), 1:400, Abcam, UK) overnight at 4° C in a humidified chamber. DAB (3,3’-diaminobenzidine) was used as a chromogenic substrate, and the sections were counterstained with hematoxylin. The IHC staining intensity was classified separately for the nucleus/nuclear membrane and cytoplasm and graded as strong (value = 3), moderate (value = 2), weak (value = 1), or absent (value = 0). In every tissue specimen, three areas with different staining intensities were selected. In each area, 100 cells were observed, and the percentage of positive cells was calculated as X_1_%. The other two values were recorded as X_2_% and X_3_%, respectively. The average of X_1_%, X_2_%, and X_3_% comprised the final staining rate. Scanning and evaluation were performed using Aperio Scanning software (Aperio XT, Leica, Germany).

### EdU assay

Hep3B cells (ATCC^®^ HB-8064) were cultured in a 6-well plate and treated with 100 μL media containing 20 μM EdU. After continuous incubation at 37° C with 5% CO_2_ for 24 h and 48 h, the cells were fixed with 4% paraformaldehyde for 15 min and incubated with 0.5% Triton X-100 in phosphate-buffered saline (PBS) for 10–15 min. Fluorescence microscopy was employed to acquire and analyze the images. All the experiments were performed at least three times, and the data were represented as mean ± standard deviation (SD).

### Transwell invasion assay

Transwell assays were performed using polyethylene terephthalate-based migration chambers and BD BioCoat Matrigel Invasion Chambers (Becton Dickinson Labware, USA). Hep3B cells were seeded on Matrigel-coated transwell inserts with 200 μL of serum-free medium. The lower chamber was filled with 500 μL medium containing 10% FBS. After incubation for 24 h, the cells remaining on the upper surface of transwell inserts were wiped with cotton wool. The invaded cells were stained with crystal violet for 10 min. Images were captured, and the cell number was counted. All the experiments were performed at least three times, and the data are expressed as mean ± SD.

### Statistical analysis

Statistical analysis was performed using SPSS version 17.0 software package (SPSS Inc., Chicago, IL, USA) and GraphPad Prism 7.0 (GraphPad Software, San Diego, CA, USA). Clinical data were presented as mean ± SD. A paired-samples t-test was performed to test the difference between tumor and paracarcinoma tissues for the replication population. Independent samples t-test was performed to test the cell proliferation and invasion rates between siRNA (siARID1A, siSENP3, and siCSMD1) and siRNC groups. Cox regression analysis was used to evaluate the prognostic value adjusted by patient sex, age, and AJCC stage (version VII). We divided the samples into two groups based on the expression level (intensity times positive expression rate) and compared the accumulated survival rate between the two groups for each gene and combination of two or three genes. A receiver operating characteristic (ROC) curve analysis was performed, and the area under the ROC curve (AUC) was calculated to evaluate the prognostic power. The Kaplan–Meier method was used to assess the survival of patients and compared using the log-rank test. *P*-value < 0.05 indicated statistical significance.

### Ethics approval

The clinical HCC samples complied with the Declaration of Helsinki 1975, revised in 2008. This study was approved by the appropriate local institutional review boards on human research at the Huazhong University of Science and Technology (IRB Number: S104). Written consent was obtained from all subjects before participation in the study.

### Availability of data and materials

The data have been deposited with links to BioProject accession number PRJNA607376 in the NCBI BioProject database (https://www.ncbi.nlm.nih.gov/bioproject/).

## Supplementary Material

Supplementary Figures
