# Correction for: Combined identification of *ARID1A*, *CSMD1*, and *SENP3* as effective prognostic biomarkers for hepatocellular carcinoma

**DOI:** 10.18632/aging.206350

**Published:** 2025-12-31

**Authors:** Yuanyuan Zhao, Bo Yang, Dong Chen, Xiaojun Zhou, Meixi Wang, Jipin Jiang, Lai Wei, Zhishui Chen

**Affiliations:** 1 Institute of Organ Transplantation, Tongji Hospital, Tongji Medical College, Huazhong University of Science and Technology, Wuhan 430030, China; 2 Key Laboratory of Organ Transplantation, Ministry of Education, Wuhan 430030, China; 3 NHC Key Laboratory of Organ Transplantation, Wuhan 430030, China; 4 Key Laboratory of Organ Transplantation, Chinese Academy of Medical Sciences, Wuhan 430030, China

**Keywords:** differentially expressed genes, characteristic molecular heterogeneity, population specific biomarkers, hepatocellular carcinoma, HBV infection

**This article has been corrected:** The authors identified an error in the assembly of Figure 7A. Specifically, the image of transwell invasion assay intended to represent the siRNC (negative control) group in the SENP3 gene panel was erroneously replaced with the image from the siRNA group. This was an oversight during the figure preparation process.

The Figure 7A SENP3 siRNC panel has been replaced by the authors using data from the original experiments. This correction does not change the conclusions of the publication.

The corrected version of **Figure 7** is provided below.

**Figure 7:**
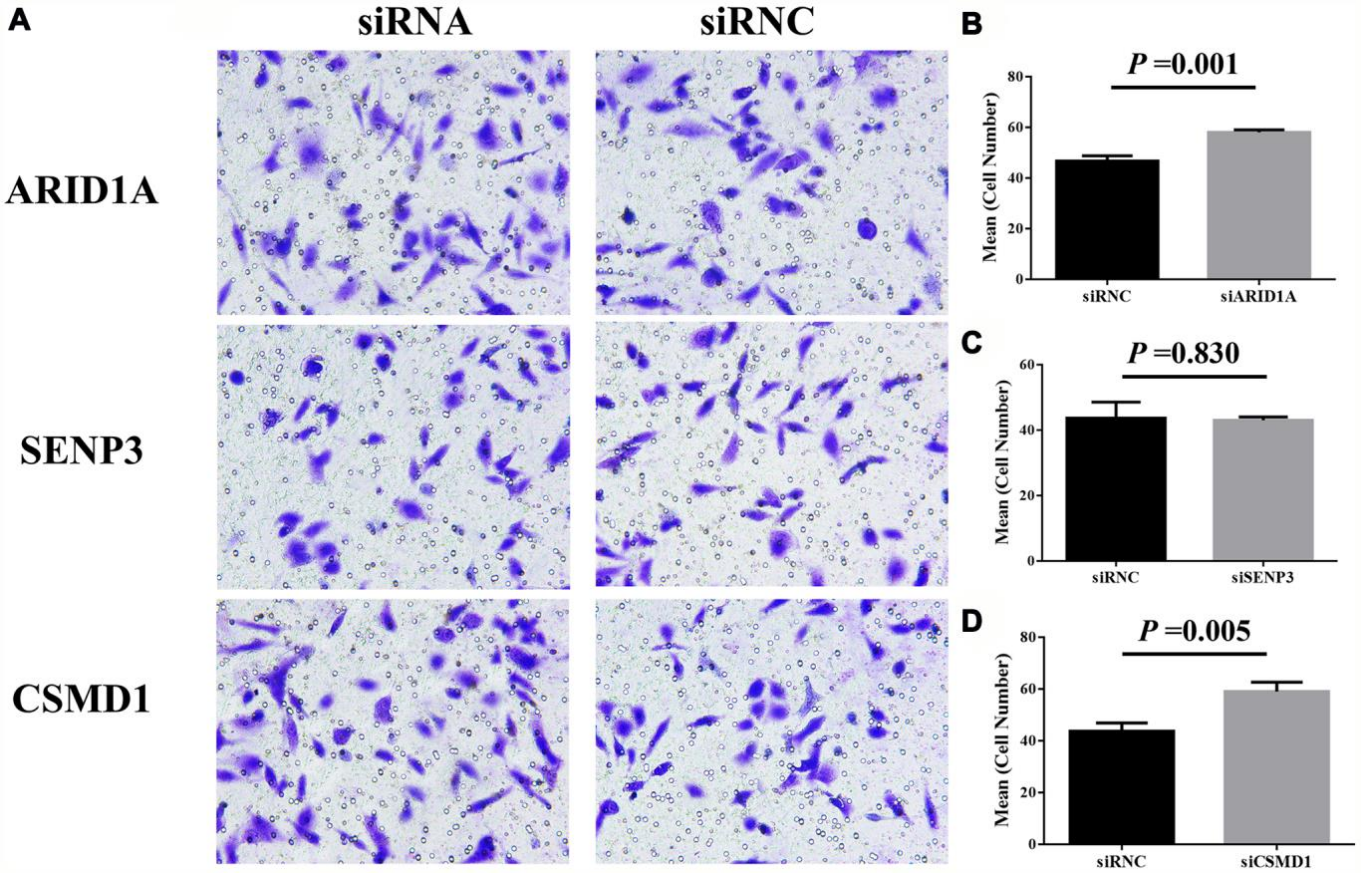
**Cell invasion after the suppression of genes, *ARID1A*, *CSMD1*, and *SENP3 *by siRNA.** The images of transwell invasion assay shown in (**A**) were scanned, quantified, and plotted in (**B**–**D**, respectively).

