# Supplementary material for: Combined identification of ARID1A, CSMD1, and SENP3 as effective prognostic biomarkers for hepatocellular carcinoma
Source: Aging (Albany NY). 2021 Feb 7;13(3):4696–712. doi: 10.18632/aging.202586 (PMC7906131; doi:10.18632/aging.202586)
Supplement: Supplementary Figures [file aging-13-202586-s001.pdf]

SUPPLEMENTARY FIGURES

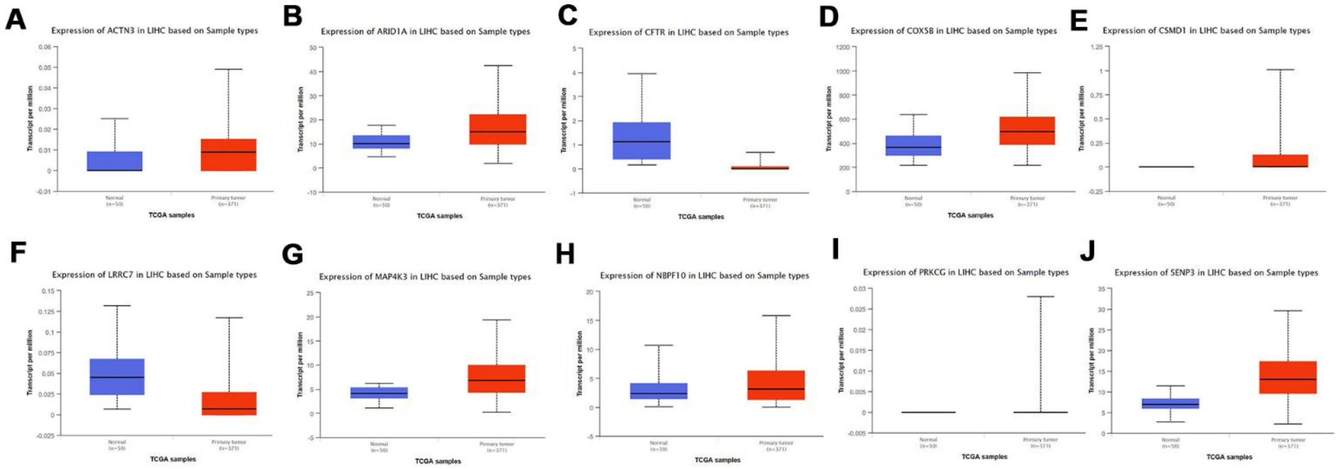

Supplementary Figure 1. Expression patterns of 11 novel genes in TCGA database. (**A**) Expression pattern of the *ACTN3* gene. *P* = 1.17E-02. (**B**) Expression pattern of *ARID1A* gene. *P* < 1.00E-12. (**C**) Expression pattern of *CFTR* gene. *P* > 0.05. (**D**) Expression pattern of *COX5B* gene. *P* = 1.62E-12. (**E**) Expression pattern of *CSMD1* gene. *P* = 1.11E-16. (**F**) Expression pattern of *LRRC7* gene. *P* value > 0.05. (**G**) Expression pattern of *MAP4K3* gene. *P* < 1.00E-12. (**H**) Expression pattern of *NBPF10* gene. *P* = 1.20E-02. (**I**) Expression pattern of *PRKCG* gene. *P* value = 1.54E-02. (**J**) Expression pattern of *SENP3* gene. *P* = 1.62E-12.

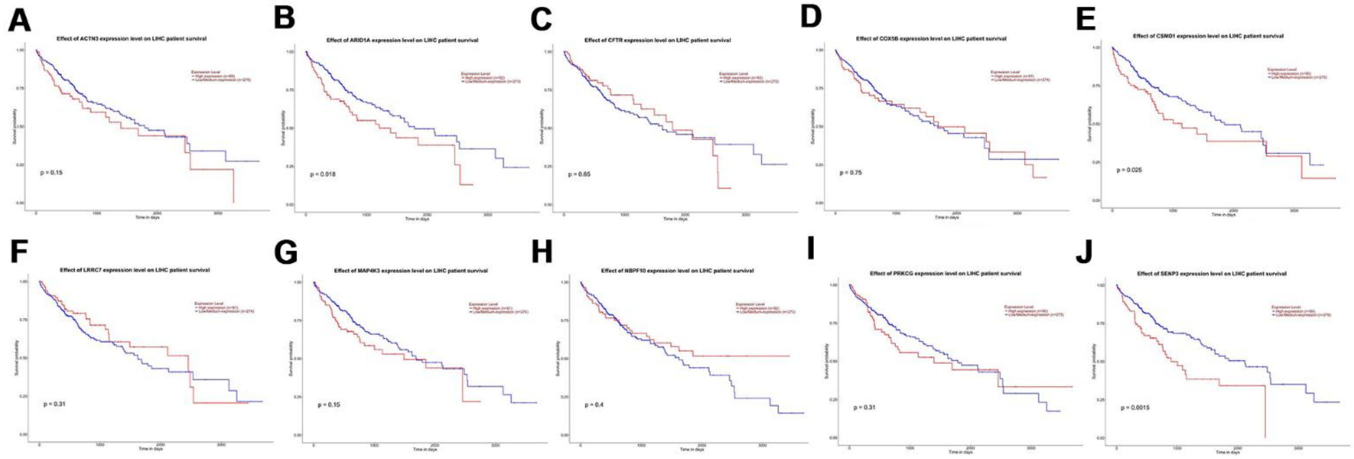

Supplementary Figure 2. Survival patterns of 11 novel genes in TCGA database. (**A**) Survival pattern of *ACTN3* gene. *P* value > 0.05. (**B**) Survival pattern of *ARID1A* gene. *P* = 0.018. (**C**) Survival pattern of *CFTR* gene. *P* > 0.05. (**D**) Survival pattern of *COX5B* gene. *P* > 0.05. (**E**) Survival pattern of *CSMD1* gene. *P* = 0.025. (**F**) Survival pattern of *LRRC7* gene. *P* > 0.05. (**G**) Survival pattern of *MAP4K3* gene. *P* > 0.05. (**H**) Survival pattern of *NBPF10* gene. *P* > 0.05. (**I**) Survival pattern of *PRKCG* gene. *P* > 0.05. (**J**) Survival pattern of *SENP3* gene. *P* = 0.0015.
